# A murine cytomegalovirus cell cycle regulator (m54.5p) evolved within the conserved viral DNA polymerase gene

**DOI:** 10.1371/journal.ppat.1013424

**Published:** 2026-05-22

**Authors:** Yan Zheng, Ivana Bertovic, Xiangming Han, Thomas Hennig, Adam W. Whisnant, Stephanie Lamer, Andreas Schlosser, Vanda Juranic Lisnic, Penelope Kay-Fedorov, Lars Dölken, Manivel Lodha

**Affiliations:** 1 Institute for Virology, Julius-Maximilians-University Würzburg, Würzburg, Germany; 2 Institute for Virology, Hannover Medical School, Hannover, Germany; 3 Faculty of Medicine, Center for Proteomics, University of Rijeka, Rijeka, Croatia; 4 Rudolf Virchow Center, Center for Integrative and Translational Bioimaging, Julius-Maximilians-University Würzburg, Würzburg, Germany; 5 Cluster of Excellence RESIST (EXC2155), Hannover Medical School (MHH), Hannover, Germany; 6 The Rockefeller University, New York, New York, United States of America; Cardiff University, UNITED KINGDOM OF GREAT BRITAIN AND NORTHERN IRELAND

## Abstract

Ribosome profiling (Ribo-seq) and transcription start site profiling recently unveiled hundreds of novel viral gene products in lytic murine cytomegalovirus (MCMV) infection. One of these is the highly expressed m54.5 open reading frame (ORF) located within the highly conserved viral DNA polymerase locus (M54). Here, we show that m54.5 encodes a nuclear protein (m54.5p) that contributes to cell cycle regulation during MCMV infection. m54.5p interacts with the anaphase-promoting complex/cyclosome (APC/C) and protein phosphatase-6 (PP6). Ectopic m54.5p expression resulted in nuclear accumulation of PP6C and multiple APC/C subunits, accompanied by increased nuclear levels of the APC/C substrates. Accordingly, ectopic m54.5p expression resulted in G1 cell cycle arrest in nocodazole mitotic arrest and serum starvation assays. While an m54.5p-null mutant virus replicated with wild-type kinetics *in vitro*, it was mildly attenuated in mouse lungs by 14 days post-infection. Our findings highlight the surprising plasticity of herpesvirus genomes, facilitating the evolution of a > 200 aa viral open reading frame within the highly conserved viral DNA polymerase gene locus.

## Author summary

Viruses commonly hijack host cell processes to support their replication. Using advanced sequencing approaches, we recently discovered a novel gene, *m54.5*, encoded within the highly conserved viral DNA polymerase locus of the murine cytomegalovirus (MCMV) genome. This gene encodes a nuclear protein, m54.5p, abundantly expressed early during lytic infection. Here, we show that m54.5p interacts with host cell cycle regulators, the anaphase-promoting complex/cyclosome (APC/C) and protein phosphatase-6 (PP6), and arrests cells in G1 phase, blocking progression into S phase and cellular DNA replication during lytic MCMV infection. Our findings show that m54.5p induces a functionally inactive APC/C complex, leading to nuclear accumulation of APC/C substrates. In contrast to the unrelated UL21a protein of human cytomegalovirus, which promotes G1 cell cycle arrest by degrading core APC subunits, m54.5p induces a functionally inactive APC/C complex, illustrating convergent evolution of cytomegalovirus cell cycle regulators. While m54.5p was dispensable for efficient virus replication in vitro, its relevance during productive infection in mice became apparent by 14 days post-infection in the lungs. Our findings reveal unexpected plasticity of herpesvirus genomes, enabling the evolution of new viral regulators even within one of the most highly conserved viral gene regions.

## Introduction

Human cytomegalovirus (HCMV) is a double-stranded DNA virus with a genome of ~240 kb, classified within the beta-herpesvirus subfamily [1,2]. It establishes lifelong persistent infections in humans [3]. Globally, HCMV infection affects about 60% to 90% of the population, with higher prevalence in developing countries [4]. While mostly asymptomatic in healthy individuals, HCMV poses significant risks to immunocompromised patients, transplant recipients, and neonates [5]. In neonates, congenital HCMV infection is the leading cause of intellectual disabilities and sensorineural hearing loss [6]. The virus exhibits strict species specificity, which presents challenges in studying its biology and immune evasion mechanisms [7].

Murine cytomegalovirus (MCMV) shares significant similarities with HCMV, making it a valuable model for studying HCMV pathogenesis and immune evasion [8]. Both viruses have evolved a plethora of different mechanisms to invade host cells and establish infection, heavily relying on the host cellular machinery. Their genome replication is closely linked to the host cell cycle, which comprises the mitotic (M), growth 1 (G1), DNA synthesis (S), and growth 2 (G2) phases in actively dividing cells [9–12]. Both viruses encode their own DNA polymerases (UL54 in HCMV; M54 in MCMV), and replication factors for viral DNA synthesis, but rely on cellular resources, such as deoxynucleotides (dNTPs) and replication factors [13,14]. As most infected host cells are in a quiescent cell cycle state (G0) with a low deoxyribonucleotide pool, cytomegaloviruses evolved multiple viral proteins to prompt re-entry of their target cells into the cell cycle, subsequently establishing an atypical S-like phase, also referred to as ‘pseudo-S-phase’, by arresting the cell cycle at the G1/S transition. Thereby, they promote viral genome replication while efficiently inhibiting cellular DNA synthesis [11,15–17].

Over the years, numerous mechanisms employed by both murine cytomegalovirus (MCMV) and human cytomegalovirus (HCMV) to induce the G1/S phase arrest have been documented [10–12,18,19]. Both viruses utilize similar pathways, including modulation of cell cycle regulatory Rb-E2F transcription factors, the p53-p21 checkpoint, the DREAM complex, and targeting cyclin A and E kinases and SAMHD1 proteins [20–28]. Accordingly, the MCMV kinase M97 and its HCMV homolog, UL97, modulate the DREAM complex by targeting the LIN54 and LIN52 subunits, respectively [27,28]. However, there are substantial differences in the mechanisms by which these two viruses regulate the cell cycle. MCMV M117 targets the E2F 1–5 transcription factors, whereas its HCMV counterpart, UL117, specifically targets the mini-chromosome maintenance (MCM) complex, most notably MCM2 and MCM4, blocking their accumulation and chromatin loading to inhibit host DNA replication [18,29]. The HCMV UL82 gene product, pp71, drives quiescent cells into the cell cycle through its LxCxE motif and promotes proteasome-dependent degradation of hypophosphorylated retinoblastoma (Rb) protein and its family members, p107 and p130, an interaction not conserved in the MCMV homologs, M82 and M83 [30]. HCMV pp150 (pUL32) contains an RXL motif that binds cyclin A, restricting IE gene expression in S/G2 phases and preventing mitotic cell death in cooperation with pUL21a-mediated cyclin A degradation [25,31]. In contrast, MCMV lacks this regulation in its pp150 homolog but utilizes M97, which binds Cyclin A to alter its function and localization [27].

The APC/C complex is an evolutionarily conserved E3 ubiquitin ligase that controls cell cycle progression by mediating the spatiotemporal ubiquitination and degradation of key regulatory proteins [2,32]. The pUL21a, which is conserved only in primate CMVs, binds directly to APC/C, specifically targeting its subunits APC4 and APC5 for degradation. Thereby, pUL21a triggers destabilization and dissociation of the APC/C, disrupting its E3 ubiquitin ligase activity. The resulting accumulation of APC/C substrates, such as geminin, facilitated cell cycle arrest at the G1/S phase transition and, consecutively, productive viral replication [32].

**Fig 1:**
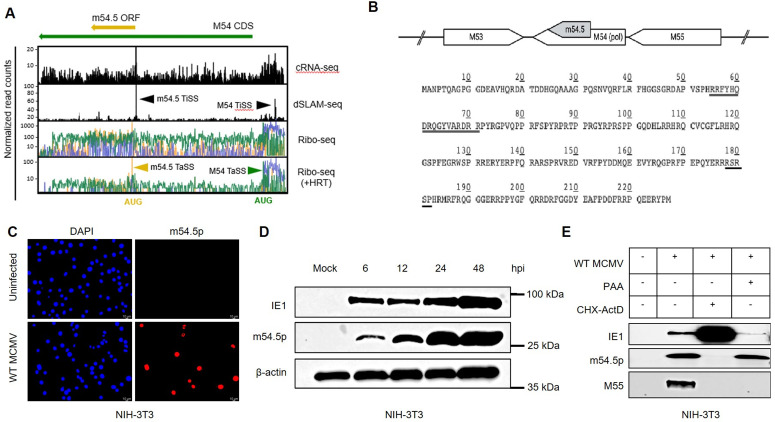
m54.5 encodes a nuclear m54.5 (m54.5p) expressed with early kinetics. **A**. Schematic representation of the annotated viral transcripts and ORFs in the MCMV M54/m54.5 locus. Normalized read counts (y-axis) for ribosome profiling (Ribo-seq), as well as transcription start site profiling data (dSLAM-seq and cRNA-seq) (8), aggregated across a time course during MCMV lytic infection in NIH-3T3 fibroblasts, are shown. dSLAM-seq transcription start sites (TiSS) are depicted in black. Translation start sites (TaSS) are depicted in green and yellow for the M54 and m54.5 ORFs, respectively. Ribosome profiling data (including translation start site enrichment using harringtonine – HRT) are represented on a logarithmic scale, cRNA-seq and dSLAM-seq are represented on a linear scale. The schematic representation is based on data published by Lodha et al (8). **B**. Schematic diagram of the genomic region covering M53 to M55, including the amino acid sequence of the m54.5 ORF (227 aa). The bipartite NLS and ^178^RSRSP^182^ sequence is underlined. **C**. Uninfected NIH-3T3 cells and NIH-3T3 cells infected with WT MCMV for 24 hours were subjected to immunostaining for m54.5p. DAPI was used for nuclear counterstaining. Data from two biological replicates are shown; an independent replicate is shown in S4 File. Time-course immunoblot profiling of m54.5p during MCMV lytic infection in NIH-3T3 fibroblasts. Mock represents uninfected cells. Immunoblotting was performed for MCMV IE1, m54.5p, and β-actin (loading control). Data of a second, independent biological replicate is shown in S5 File. Chemical inhibitor treatment reveals that m54.5p is expressed with early kinetics. Immunoblotting of m54.5p, IE1, and M55 expression for NIH-3T3 cells infected with MCMV without chemical inhibitors, PAA, or CHX-ActD reversal is shown. Two biological replicates were performed. Data from the second replicate is shown in S4 File. PAA: Phosphonoacetic acid, CHX: Cycloheximide, ActD: Actinomycin D.

More recently, we provided a comprehensive reannotation of the MCMV genome, studying lytic MCMV gene expression in NIH-3T3 murine fibroblasts. Using ribosome profiling (Ribo-seq) and transcription start site profiling (cRNA-seq and dRNA-seq), we identified and annotated hundreds of novel open reading frames (ORFs) and the viral transcripts from which they are expressed. The majority of so far unknown viral ORFs included short, upstream and upstream-overlapping ORFs (uORFs and uoORFs), consistent with recent findings for several other herpesviruses, including HCMV [33], HSV-1 [34], KSHV [35], and EBV [36]. However, we also identified so far unknown large viral ORFs of >100 amino acids (aa) in size, such as *m54.5* (227 aa), which is located fully within the highly conserved viral DNA polymerase (M54) ORF (Fig 1). *m54.5* was excluded in previous in-silico predictions due to its 100% overlap with the M54 ORF. Interestingly, *m54.5* is absent in other cytomegaloviruses and thus appears to have evolved specifically in MCMV.

Here, we characterized the *m54.5* gene product (m54.5p). We show that m54.5p contributes to the virus-induced cell cycle arrest at the G1/S transition by inducing a catalytically inactive APC/C complex and nuclear accumulation of its cell cycle substrates. While dispensable for productive virus replication *in vitro*, m54.5p is required for efficient virus replication in Balb/c mice. Most importantly, however, our findings highlight a surprising plasticity of a herpesvirus genome, facilitating the evolution of a 227 aa ORF within one of the most highly conserved regions of herpesvirus genomes.

## Results

### m54.5 encodes a nuclear protein (m54.5p) expressed with early kinetics

We previously conducted a comprehensive reannotation of the MCMV gene products expressed during lytic infection of murine NIH-3T3 fibroblasts based on Ribo-seq and transcriptomic analysis [8]. In this work, we identified a novel ORF (m54.5), internal to the coding sequence of the MCMV DNA polymerase gene (M54), expressed at higher levels than the viral DNA polymerase itself **(**Fig 1A). BLAST analysis indicated that the m54.5 ORF is 100% conserved in all known MCMV strains without a single nucleotide mutation (S1 File). The absence of any significant stretch of codons without a stop codon in any other frame of M54 homologues in other cytomegaloviruses excludes conservation of m54.5 in other cytomegaloviruses and thus specifically evolved in MCMV (S2 File). Moreover, m54.5 lacks amino acid homology with other CMV species (including rat and human CMV) and with UNIPROT cellular proteins (S3 File). Subcellular localization prediction using PSORT II indicated a strong classical bipartite nuclear localization signal (NLS) (Fig 1B), and m54.5p also contained an ^178^RSRSP^182^ sequence, previously shown to regulate nuclear localization of the RBM20 splicing factor [37]. We hence decided to study the function of m54.5p, given its high translational activity across the locus and its interesting association with the essential viral polymerase (M54).

As the *m54.5* ORF cannot be tagged without affecting the coding sequence of the viral DNA polymerase, we generated a monoclonal mouse antibody against m54.5p, which we found to be highly specific for m54.5p and suitable for both immunofluorescence analysis (IF) and immunoblotting. IF of MCMV-infected cells at 24 hours post-infection (hpi) confirmed nuclear localization of m54.5p (Fig 1C and S4 File). Next, we studied m54.5p expression throughout lytic MCMV infection by immunoblotting. We found that m54.5p is already well expressed by 6 hours post-infection (hpi) and accumulates to high levels at late times of infection (Fig 1D and S5 File). Chemical inhibition of viral DNA synthesis using phosphonoacetic acid (PAA) had no effect on m54.5p expression, consistent with kinetics typical of viral early or immediate-early genes. To further characterize the m54.5p expression profile, we performed Cycloheximide (CHX)–Actinomycin D (ActD) reversal experiments. In this approach, CHX is first applied to block *de novo* viral protein synthesis while permitting transcription of immediate-early (IE) genes. Subsequent CHX removal and ActD addition allow translation of newly transcribed viral IE mRNAs. These experiments confirmed that m54.5p is expressed with early kinetics and validated the expression of the m54.5 protein (Fig 1E and S4 File).

### An m54.5p-null mutant virus exhibits comparable growth and replication kinetics to wild-type MCMV *in vitro*

**Fig 2:**
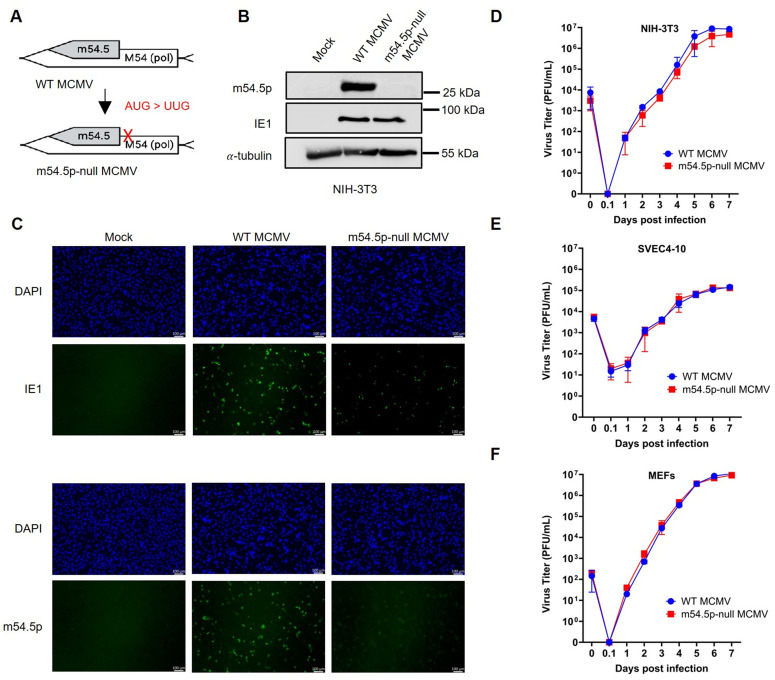
An m54.5p-null virus shows no attenuation *in vitro.* **A**. Schematic representation of the mutagenesis approach for generating the m54.5p-null mutant. **B**. NIH-3T3 cells infected for 24 h with MCMV wild-type (WT), its m54.5p-null mutant, or mock. Protein expression of m54.5p, IE1, and α-tubulin (loading control) was quantified by immunoblot. An independent biological replicate is shown in S4 File. NIH-3T3 cells infected for 24 h at an MOI of 1 with the indicated viruses were immunoblotted for m54.5p and MCMV IE1. DAPI was used to stain cell nuclei. An independent replicate is shown in S5 File. Growth curves for wild-type (WT) MCMV or its m54.5p-null mutant initiated at an MOI of 0.1 on **(D)** NIH-3T3, **(E)** SVEC4-10 endothelial cells, and (**F**) primary murine embryonic fibroblasts (MEF). Each growth curve depicts the combined data of two independent growth curve experiments. The sample labeled “0.1” represents the supernatant collected immediately after viral adsorption, followed by washing with 1x PBS.

To assess the importance of m54.5p for productive infection, we constructed an MCMV mutant (m54.5p-null) on the pSM3fr backbone (*en passant* mutagenesis [38]) by mutating the start codon of m54.5 (AUG > UUG) without altering the M54 amino acid sequence (Fig 2A). The newly generated mutant showed no m54.5p expression by IF and immunostaining (Fig 2B-2C and S4 and S5 Files). It is important to note that there are no downstream in-frame AUGs within 468 nt that would enable translation initiation and expression of a truncated m54.5p. Multi-step growth curve analysis on NIH-3T3 fibroblasts, SVEC4–10 endothelial cells, and primary murine embryonic fibroblasts (MEF) demonstrated that the m54.5p-null mutant replicated with kinetics comparable to wild-type (WT) MCMV (Fig 2D-2F). We conclude that m54.5p is dispensable for viral replication and growth *in vitro* in major MCMV target cell types.

### m54.5p interacts with components of the PP6 and APC/C complex

**Fig 3:**
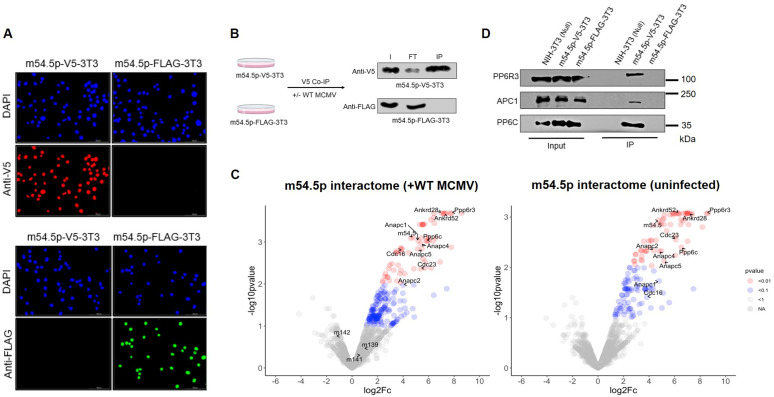
m54.5p interacts with components of the PP6 and APC/C complex. **A.** m54.5p-V5-3T3 and m54.5p-FLAG-3T3 cells were stained for V5- and FLAG-tag epitopes. DAPI was used as a nuclear counterstain. **B.** MCMV-infected or uninfected m54.5p-V5-3T3 and m54.5p-FLAG-3T3 cells were subject to V5 Co-IP. Two biological replicates were performed. Representative Western blots for the V5 Co-IP are shown. Efficiency is shown by enrichment of V5-tagged m54.5p in the IP sample. I: Input m54.5, FT: Flow-through after IP; IP: Co-IP Output. Three independent replicates are shown in S4 File. Volcano plots showing m54.5p interactome analysis by Co-IP LC-MS conducted for V5 Co-IP (+/- WT MCMV infection). Key components of the APC/C and PP6 complexes are indicated. The y-axis represents -log10 adjusted p-values for each interaction, while the x-axis depicts the log2 fold change representing the enrichment of V5 over FLAG. Colors represent varying degrees of statistical significance. Components of the PP6 (PP6R3, PP6C, ANKRD 28 and 52) and APC/C complex (APC1, APC4, and APC5) are indicated. Each volcano plot depicts the data from three biological replicates per condition. **D.** Immunoblot validation of m54.5p interactors (PP6R3, PP6C, APC1) by V5 Co-IP as performed in **B**. Data from independent biological replicates are shown in S4 File.

As m54.5p lacks homology to any known cellular or viral proteins, we resorted to functionally characterizing m54.5p by studying its interactome using co-immunoprecipitation (Co-IPs) combined with LC-MS. To this end, we generated polyclonal NIH-3T3 cell lines constitutively expressing m54.5p, C-terminally tagged with either a V5- or a FLAG-tag (m54.5p-V5-3T3 and m54.5p-FLAG-3T3) (Fig 3A). Expression of FLAG-tagged m54.5p served as an ideal negative control for the V5-tag Co-IP LC-MS. It ensured that any qualitative or quantitative changes in the cellular proteome due to ectopic m54.5p expression were accounted for. After validating the cell lines and analyzing V5 Co-IP efficiency (Fig 3B and S4 File), we performed Co-IP LC-MS for both cell lines under both uninfected and MCMV-infected (24 hpi) conditions (three biological replicates per condition). The m54.5p interactomes were plotted as volcano plots showing the average enrichment as fold change against the respective p-values. All interactors are listed in S1 Table, filtered by their p-values. Notably, three distinct complexes, including the small subunit of the mitochondrial ribosomal proteins (MRPS), the protein phosphatase-6 complex (PP6), and the anaphase-promoting cyclosome complex (APC/C or ANAPC) emerged as strong interactors of m54.5p. No enrichment of viral proteins was detected in the MCMV-infected samples, suggesting that m54.5p does not associate with additional viral proteins to exert its function (Fig 3C). As m54.5p is a nuclear protein, we focused on the nuclear protein complexes PP6 and APC/C. Importantly, V5 Co-IP and immunoblotting confirmed the interactions of m54.5p with the top-interacting components of the respective complexes, namely APC1 (ANAPC1), PP6, and PP6R3 (Fig 3D and S4 File).

### m54.5p expression results in cell cycle arrest at the G1/S interphase

**Fig 4:**
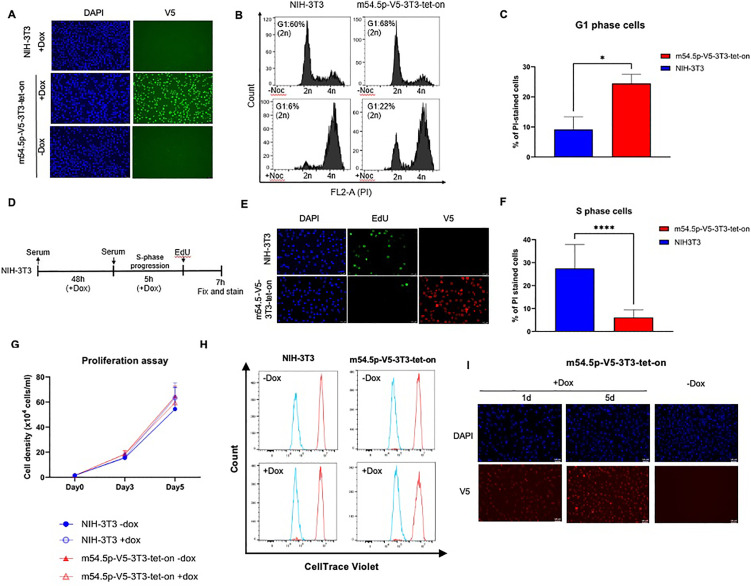
m54.5p expression results in cell cycle arrest at the G1/S interphase. **A.** m54.5p-V5-3T3-tet-on and NIH-3T3 cells treated with or without 1 μg/ml doxycycline (Dox) for 24 h were stained for V5. DAPI was used for nuclear counterstaining. **B.** m54.5p-V5-3T3-tet-on and NIH-3T3 cells were treated with 1 μg/ml doxycycline (Dox) for 24 h, followed by +/- nocodazole (Noc) treatment for 16 h (0.8 μg/ml) in the presence of doxycycline. Cells were harvested, fixed, and stained for DNA content via propidium iodide staining in the presence of RNase A using flow cytometry. The percentage of cells in G1 (2n DNA content) is shown for a representative of three biological replicates. An independent replicate is shown in S5 File**.** Bar graph showing the percentage of cells in G1 (2n DNA content) from B. Statistical significance was determined using the two-tailed parametric t-test * (p < 0.05), n = 2. **D.** Schematic of the serum starvation S-phase assay. **E.** m54.5-V5-3T3-tet-on and NIH-3T3 cells were subject to the serum starvation S-phase assay and stained for V5 and newly synthesized DNA via EdU labelling. EdU incorporation was visualized by attaching an azide (conjugated with Alexa fluor 488 dye) via click chemistry. DAPI was used for nuclear counterstaining. An independent replicate is shown in S5 File. Bar graph showing the percentage of S-phase cells from E. Data of three images from three biological replicates (n = 9) are shown. **, p < 0.0001. Statistical significance was determined using the two-tailed parametric t-test. **G**. Proliferation of m54.5-V5-3T3-tet-on and NIH-3T3 cells: 30,000 cells per well of a 6-well dish were seeded (day 0) and allowed to grow for up to 5 days in the presence or absence of doxycycline. The number of cells per well obtained after 3 and 5 days from each condition is shown. Combined data from two independent biological replicates, with six individual wells per condition and time point, are shown. **H**. Proliferation of m54.5-V5-3T3-tet-on and NIH-3T3 cells labeled with CellTrace Violet at day 0 and analyzed by FACS at day 5. The leftward shift of the peaks indicates homogenous cell proliferation in the culture irrespective of m54.5p expression. **I**. Immunostaining of m54.5-V5-3T3-tet-on after 1 and 5 days of doxycycline exposure depicted strong conditional expression of m54.5p. A representative experiment of two biological replicates is shown. An independent replicate is shown in S5 File.

The APC/C E3 ubiquitin ligase complex is a master regulator of the cell cycle. The PP6 complex regulates mitotic spindle formation and transition into S phase [39], among other functions. We thus asked whether m54.5p expression affected cell cycle progression. As the m54.5p interactome did not differ between ectopic expression and MCMV infection, we studied the role of ectopic m54.5p expression on the cell cycle to avoid confounding effects of other MCMV cell cycle regulators [11,12,18]. Of note, stable ectopic m54.5p expression in NIH-3T3 cells, achieved by lentiviral transduction, facilitated the generation of a polyclonal cell line. Accordingly, ectopic m54.5p expression was not detrimental to cell cycle progression and cell viability. To exclude adaptation of these cells to ectopic m54.5p expression, we generated polyclonal NIH-3T3 with conditional (doxycycline-inducible) m54.5p expression with a C-terminal V5-tag by lentiviral transduction. Following cell line validation by IF (Fig 4A), we performed nocodazole mitotic arrest assays by treating doxycycline-pre-treated NIH-3T3 and m54.5p-V5-3T3-tet-on with nocodazole for 16 hours to cause G2/M phase arrest consistent with tetraploid 4n DNA content. Cells were fixed and stained for DNA content using propidium iodide. Interestingly, while the majority of cells treated with nocodazole arrested at G2/M phase (4n), m54.5p-V5-3T3-tet-on cells showed a significantly greater number of cells arrested at G1 phase (2n) (Fig 4B-4C and S5 File). To investigate the potential impact of G1 cell cycle arrest on S-phase progression, we conducted a serum-starvation S-phase assay (Fig 4D). Briefly, NIH-3T3 and m54.5p-V5-3T3-tet-on cells, pre-treated for 16 h with doxycycline, were synchronized at the G0 phase for 48 h by serum starvation. Subsequently, the medium was replaced with fresh serum-containing medium for 5 h to induce cell cycle re-entry. Active DNA replication was subsequently measured using 5-ethynyl-2-deoxyuridine (EdU) labeling. Doxycycline-treated NIH-3T3 cells exhibited a significantly higher proportion of S-phase cells compared to similarly treated m54.5p-V5-3T3-tet-on cells (Fig 4E-4F and S5 File). Finally, we studied the impact of conditional m54.5p expression on cell proliferation by plating 30,000 m54.5p-V5-3T3-tet-on cells or their parental NIH-3T3 cells per well in a 6-well dish and monitoring proliferation over the next 5 days. In parallel, we labeled the cells with CellTrace Violet dye to monitor cell division at the single-cell level by flow cytometry. Finally, we also analyzed m54.5p expression by immunostaining after 1 and 5 days of doxycycline treatment. Consistent with the viability of stable m54.5p expression, doxycycline-induced m54.5p expression did not result in a notable difference in the number of cells obtained after 5 days (Fig 4G) or in proliferation rates, as assessed by CellTrace Violet labeling (Fig 4H). Moreover, the majority of m54.5p-V5-3T3-tet-on still showed strong m54.5p expression after 5 days of doxycycline exposure (Fig 4I and S5 File). We conclude that m54.5p acts as a fine-tuner of cell division rather than a strict cell cycle arrest protein (like M97).

### m54.5p induces nuclear accumulation of APC/C components and cell cycle substrates

**Fig 5:**
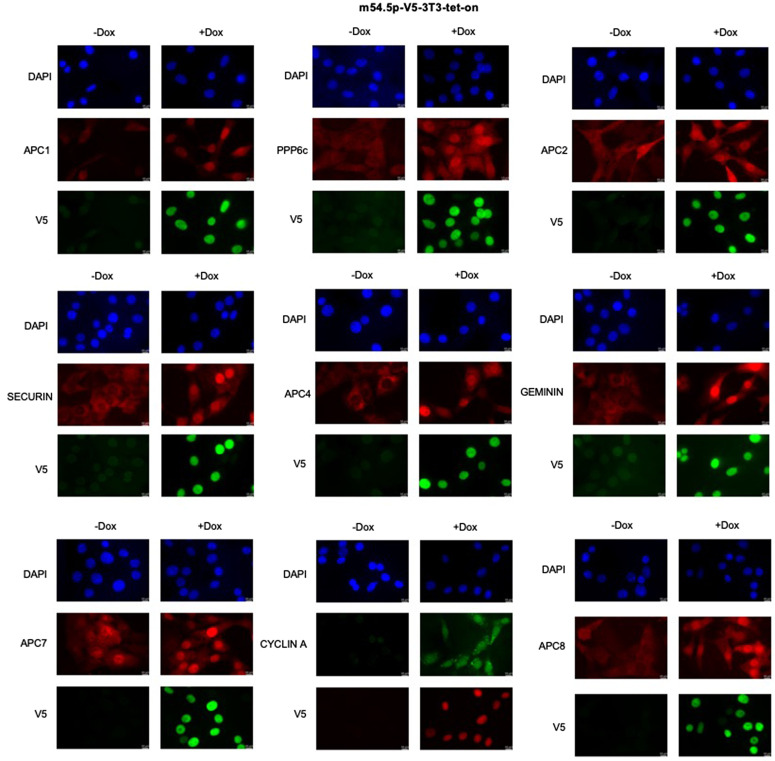
m54.5p induces nuclear accumulation of APC/C components and cell cycle substrates. Confocal microscopy of polyclonal NIH-3T3 cells engineered for doxycycline-induced expression of a V5-tagged m54.5 protein and control NIH-3T3 cells. Cells were exposed to doxycycline for 24 h prior to immunostaining, as indicated (+Dox vs -Dox). DAPI was used to counterstain cell nuclei. Representative pictures of key APC/C components (APC1, APC2, APC4, APC7, and APC8), the catalytic PP6 component PP6C, and the three APC/C substrates geminin, securin, and Cyclin A are shown. Control immunostaining for APC/C components, PP6C, and the cell cycle substrates is shown in the S6 File.

Cell cycle progression is accompanied by altered levels and subcellular localization of major cell cycle regulators and their substrates. To understand how m54.5p fine-tunes the cell cycle through its interaction with the APC/C and PP6 complex, we studied changes in protein localization and abundance by immunostaining and confocal microscopy for both m54.5p-V5-3T3-tet-on and NIH-3T3 cells with or without doxycycline pretreatment. Intriguingly, induction of m54.5p resulted in nuclear accumulation of all APC/C components we probed for, including APC1, APC2, APC4, APC7, and APC8, as well as the protein phosphatase-6 catalytic domain (PP6C) (Fig 5 and S6 File). Moreover, we observed strong nuclear accumulation of three common APC/C cell cycle substrates, namely geminin, securin, and Cyclin A, consistent with inhibition of APC/C ubiquitin ligase activity (Fig 5 and S6 File). To complement these immunofluorescence findings, we performed Western blot analysis on whole-cell lysates from m54.5p-V5-3T3-tet-on cells with or without doxycycline induction. In contrast to the striking changes in subcellular localization, protein expression levels of APC1, APC2, APC4, APC7, geminin, PP6C, and Cyclin A remained largely unchanged upon m54.5p induction. Securin and APC8 remained undetected with the available antibodies under these conditions (S7 File). APC/C activity during the cell cycle is well described to be regulated by hyperphosphorylation of APC/C subunits, notably APC1 and APC3 [40]. Our findings are consistent with a model in which m54.5p functions as an adaptor that recruits PP6 from the cytoplasm to the nucleus to dephosphorylate APC/C and thus inhibit APC/C activity, thereby explaining the observed arrest at the G1/S phase border.

### m54.5p mutant MCMV exhibits attenuation *in vivo*

**Fig 6:**
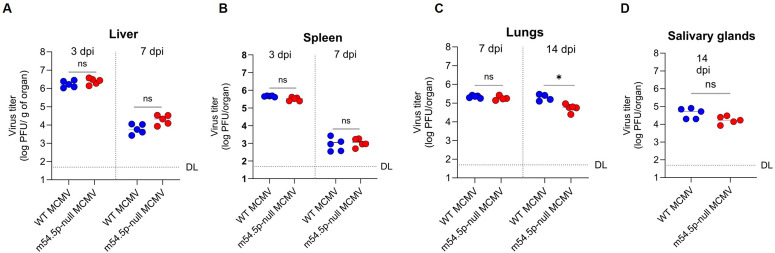
m54.5p mutant MCMV exhibits attenuation *in vivo* and in primary mouse cells. Balb/c mice were infected intravenously (i.v.) with 2x10^5^ plaque-forming units (PFU) of either wild-type MCMV or *m54.5mut* MCMV. At 3, 7, and 14 days post-infection (dpi), mice were sacrificed, and organs were harvested and subjected to virus titration on MEF. Each dot represents the data of an individual mouse. Median virus titer per condition is indicated by the horizontal lines. Significant differences were determined using unpaired Mann-Whitney t-tests. * p-value (<0.05).

To assess the role of m54.5p in acute MCMV infection of mice, we intravenously (i.v.) infected Balb/c mice with 2x10^5^ plaque-forming units (PFU) of either wild-type MCMV or its m54.5p-null mutant. No significant differences in virus titers were observed at 3 and 7 days post infection (dpi) in liver, lung, and spleen in two independent experiments (Fig 6). By 14 dpi, however, the m54.5p-null mutant was significantly attenuated (p = 0.0171) by ≈3-fold in lungs, and showed a trend towards attenuation in salivary glands (Fig 6). We conclude that m54.5p is important for productive MCMV infection by 14 days post infection.

## Discussion

In this study, we characterized the MCMV m54.5 protein, encoded by a previously unannotated ORF located within the viral DNA polymerase gene (M54). We found that m54.5p is a nuclear viral early protein that interacts with key cell cycle regulators, including the anaphase-promoting complex/cyclosome (APC/C), protein phosphatase 6 (PP6), and induces G1/S phase arrest in murine fibroblasts.

The APC/C E3 ubiquitin ligase is a master regulator of cell cycle progression [41–43]. It controls the degradation of key cyclins and cell cycle inhibitors, e.g., Cyclin A, Cyclin B, securin, and geminin, to ensure proper mitotic exit and G1 phase maintenance [44–46]. Interestingly, the HCMV UL21a protein also interacts with the APC/C complex and degrades core APC/C subunits, thereby inhibiting APC/C activity. In addition, UL21a induces G1 arrest by degrading cyclin A independent of APC/C targeting [24]. While MCMV lacks a pUL21a homolog, our findings indicate that m54.5p evolved to functionally converge on the APC/C complex.

Doxycycline-induced ectopic expression of m54.5p resulted in a strong nuclear accumulation of the APC/C complex, the PP6 catalytic subunit PP6C, and canonical APC/C substrates securin, geminin, and Cyclin A. To determine whether this nuclear accumulation was accompanied by changes in total protein abundance, we performed Western blot analysis on whole-cell lysates from m54.5p-V5-3T3-tet-on cells with or without doxycycline pre-induction. Interestingly, total expression levels of APC1, APC2, APC4, APC7, geminin, PP6C, and Cyclin A remained largely unchanged upon m54.5p expression. Securin and APC8 could not be reliably detected under these conditions. The nuclear accumulation of APC/C, together with the nuclear accumulation of three major APC/C substrates (geminin and securin are classical signatures of APC/C inhibition), indicates that m54.5p induces a catalytically inactive APC/C complex rather than inducing APC/C degradation like pUL21a [24]. Importantly, the unchanged total protein levels of these APC/C components and substrates argue that m54.5p primarily alters their subcellular localization rather than driving protein turnover, supporting a model of functional inhibition rather than proteolytic downregulation. The accumulation of securin, an APC/C Cdc20 substrate at metaphase–anaphase, argues for direct APC/C inhibition rather than a purely downstream consequence of cell cycle perturbation [47,48]. It is important to note that APC/C activation is governed by mitotic hyperphosphorylation (e.g., CDK1-dependent phosphorylation promoting Cdc20 binding and APC/C activity [48–50]). Moreover, dephosphorylation by cellular phosphatases, such as PP2A, can inhibit APC/C ubiquitin ligase activity [51]. In combination with the nuclear relocalization of PP6C by m54.5p, we propose a model in which m54.5p acts as an adaptor that recruits PP6C from the cytoplasm to the nucleus to promote APC/C dephosphorylation and functional inhibition. While protein phosphatase 2A (PP2A) and not PP6C has been shown to inhibit APC/C ubiquitinylase activity by dephosphorylation [51,52], herpesviruses commonly redirect cellular enzymes to new cellular targets to aid their means. Validation of the proposed model will require detailed phosphoproteomics analysis to map dephosphorylation of APC/C subunits, biochemical assays showing PP6C-dependent dephosphorylation of APC/C (or its subunits), and interaction/rescue experiments (e.g., co-IP assays and catalytic dead or knockdown/rescue studies). However, our present data support the observed cell cycle effects of m54.5p and highlight both interesting commonalities and differences between APC/C manipulation by MCMV (m54.5p) and HCMV (UL21a).

PP6C has been implicated in mitotic spindle formation and the DNA damage response [39,53] but also dephosphorylates critical cell cycle regulators, including Aurora kinases and the retinoblastoma (Rb) protein [54–56]. Tnpo3 (Transportin-3), which is a nuclear import receptor, was a top interactor of m54.5p in our Co-IP LC-MS data (S1 Table). Hence, nuclear accumulation of PP6 may be driven by the m54.5p-Tnpo3 interaction. Given that MCMV already targets Rb-E2F pathways via M117 [18], m54.5p’s interaction with PP6 might also reinforce cell cycle arrest by modulating phosphorylation-dependent signaling. Alternatively, PP6 could influence viral replication indirectly by regulating stress responses or innate immune signaling.

Mechanistically, the resulting nuclear geminin accumulation offers a plausible explanation for the observed G1 arrest (by blocking origin licensing and G1/S progression), while increased Cyclin A levels are consistent with impaired APC/C CDH1 activity in late mitosis/G1 [46,57,58]. The latter may explain why medium starvation and timed release resulted in a much stronger enrichment of cells at the G1/S cell cycle interphase. Nevertheless, we cannot exclude that m54.5p may also increase APC/C substrate levels by other means, e.g., by recruiting PP6C to securin. As such, dephosphorylation of securin by cellular PP2A has been shown to stabilize securin and thereby increase nuclear securin levels [59]. Moreover, we would like to note that Cyclin A is subject to multiple regulatory inputs (including transcriptional and virus specific mechanisms), and that m54.5p’s effects may act in concert with other viral proteins (e.g., M97) to shape cell cycle outcomes, a pattern that could explain species specific differences between MCMV (which maintains Cyclin A levels) and HCMV (where pUL21a efficiently downregulates cyclin A) [24].

In addition to m54.5p, three other MCMV proteins (IE3, M97, and M117) have been shown to interfere with cell cycle progression, pushing and halting the infected cells in a pseudo-S-phase to aid viral DNA replication while efficiently inhibiting cellular S-phase DNA replication [11,18,27]. While individual deletion of MCMV IE3 [11], M97 [27], or M117 [18] were shown to cause pronounced defects in virus-driven G1/S regulation, m54.5p appears to provide a more modest, context-dependent contribution that refines APC/C modulation rather than serving as a primary driver. IE3 can induce G1/G2 arrest independently, M97 controls Cyclin A localization by cytosolic sequestration, and M117 regulates E2F-dependent S-phase gene expression. Of note, UL21a acts in concert with UL97 to more effectively antagonize APC/C activity [60].

Interestingly, stable ectopic m54.5p expression was viable in the m54.5-V5-3T3 cells, indicating that cell cycle arrest by m54.5p could be overcome without being detrimental to cell proliferation. However, we could not exclude the possibility that the cells adapted to constitutive m54.5p expression. Accordingly, we generated polyclonal NIH-3T3 cells with doxycycline-inducible m54.5p (m54.5p-V5-3T3-tet-on). For these cells, we observed a moderate cell cycle arrest in G1, which became substantially more prominent when cells were serum-starved prior to m54.5p expression. However, when monitoring cell proliferation rates upon doxycycline-induced m54.5p expression over five days, we did not observe notable differences in proliferation rates attributable to m54.5p expression. Strong m54.5p expression was still readily detectable after 5 days of doxycycline treatment. Moreover, tracing cell divisions using CellTrace labeling did not reveal any significant reduction in cell division rates. We conclude that m54.5p fine-tunes cell division rather than induces a strict cell cycle arrest (like M97). Cells can thus overcome the m54.5p-induced effects on APC/C and the resulting increase in APC/C substrates. While the precise role of m54.5p in MCMV-cell cycle modulation remains unclear, it likely acts as a complementary, fine-tuning regulator that influences APC/C substrate processing and localization, operating alongside stronger, non-redundant regulators (IE3, M97, M117). It thus contributes to MCMV’s robust, multilayered control of the host cell cycle, fostering efficient virus replication *in vivo* by arresting cells at the G1/S interphase to facilitate viral replication while efficiently inhibiting cellular DNA replication.

Redundancies in viral cell cycle regulators probably explain why an m54.5p-null mutant exhibited no significant replication defect in immortalized fibroblasts and endothelial cells, as well as primary murine embryonic fibroblasts. Nevertheless, the m54.5p-null mutant was modestly attenuated in the lungs of mice by 14 dpi, indicating that evolution of an additional cell cycle regulator targeting the APC/C complex provides a selective replication advantage *in vivo,* potentially in specific primary cell types or through immune evasion, as observed for M117 [18]. This highlights that the much more complex virus-host interactions *in vivo* require such functional redundancies to concertedly regulate key host pathways. This functional specificity is consistent with the lack of conservation of m54.5p. It indicates that m54.5p evolved after the other MCMV cell cycle regulators within the highly conserved M54 viral DNA polymerase.

Interestingly, co-IP MS also indicated m54.5p to interact with the small subunit of the mitochondrial ribosome (MRPS) (S1 Table). While this finding still requires experimental validation, we were intrigued to see so many mitochondrial ribosomal proteins of the small subunit enriched by m54.5p co-IP. Interestingly, Epstein-Barr virus (EBV) EBNA-6 protein shuttles MRPS18–2 into the nucleus to interfere with pRb-E2F1 complexes and promote S-phase entry [61]. However, except for MRPS18–2, which binds the retinoblastoma (Rb) pocket, competing with E2F1 and promoting S-phase entry [61], mitochondrial ribosomal proteins generally do not localize to the cell nucleus but are imported into mitochondria upon translation by cellular ribosomes. Further studies are required to assess the role of m54.5p in interacting with MRPS proteins and to study the functional consequence of this interaction.

In recent years, Ribo-seq analysis has revealed hundreds of previously unknown cytomegalovirus gene products [8,62]. Our findings explain this complexity by highlighting the surprising plasticity of cytomegalovirus genomes to evolve additional regulators (i.e., *m54.5* targeting the APC/C complex in convergent evolution to the HCMV UL21a protein) even within one of the most highly conserved viral gene regions, namely the viral DNA polymerase.

## Materials and methods

### Ethics statement

The Animal Welfare Committee at the University of Rijeka, Faculty of Medicine, and the National Ethics Committee for the Protection of Animals Used for Scientific Purposes (Ministry of Agriculture) approved all animal experiments.

### Cell culture, viruses, and infection

NIH-3T3 (ATCC CRL – 1658) Swiss murine embryonic fibroblasts were grown in DMEM (Dulbecco’s Modified Eagles’ Medium) supplemented with 10% NCS (newborn calf serum) and 100 IU/ml penicillin and 100μg/ml streptomycin. Polyclonal NIH-3T3 cells engineered to express m54.5 (constitutively or inducibly) were generated by transducing the respective lentivirus supernatants using centrifugation at 800G/30 minutes with 1μg/ml polybrene. Transduced NIH-3T3 cells were grown for three days before selection with 5 μg/ml puromycin to generate polyclonal cell lines. 293T Human embryonic kidney (HEK) epithelial cells were grown in DMEM (Dulbecco’s Modified Eagles’ Medium) supplemented with 10% FCS (Fetal calf serum) and 100 IU/ml penicillin and 100μg/ml streptomycin. Primary mouse embryonic fibroblasts (MEFs, C57BL/6J) were grown in DMEM (Dulbecco’s Modified Eagles’ Medium) supplemented with 10% FCS (Fetal calf serum), 2 mM Glutamine Stable, 100 IU/ml penicillin, and 100μg/ml streptomycin. All cells were grown at 37^o^C at 5% CO_2_ under humid conditions. All MCMV viruses in this study were generated on the pSM3fr MCMV Smith background. Crude virus stocks were generated in NIH-3T3 cells. Viruses were titrated by plaque assay on NIH-3T3 cells [63,64]. Cells were infected with MCMV at an MOI of 1 for one hour at 37^o^C at 5% CO_2_, followed by media exchange, marking the 0-hour post-infection time-point.

### Mouse infections

Mice were housed and bred under specific pathogen‐free (SPF) conditions at the Central Animal Facility, Faculty of Medicine, University of Rijeka, where they were maintained at 22°C in a 12 h light‐dark cycle, and relative humidity (40%–50%). All experiments were conducted using age‐matched adult male mice (8–12 weeks old), handled in accordance with institutional and national guidelines. The animals were infected intravenously (i.v.) with 2 × 10^5^ PFU/mouse of WT MCMV and m54.5mut MCMV diluted in pure DMEM. Organs were collected at indicated time-points after infection. Virus titers were determined on murine embryonic fibroblasts (MEFs) using standard plaque assay procedures [65].

### Virus mutagenesis

The MCMV m54.5p-null mutant (*m54.5mut*) was generated on the pSM3fr MCMV Smith backbone cloned as a bacterial artificial chromosome (BAC) in *E. coli* GS1783 using *en passant* markerless mutagenesis [66]. Briefly, a Kan^r^ marker containing PCR product flanked by homologies to the desired regions in the m54.5 locus harboring a start codon mutation (ATG > TTG) was generated using the CloneAmpHiFi PCR premix (Takara Bio. 639298) using primers 5’-TTGGTGCACTGCTTCATCGCCTCCCGGACCAGCCCAGCTTGCGTTGGGTTAGCCAACGTACAGCAGCTCCCAGCTTGCGTTGGGTTAGCCAACGTACCAGCAGCTCGGCCAGCAGAGAGCGATAGGGATAACAGGGTAATCGATTT-3’ and 5’-TTTGTGCGGGAGAACGTACATCGCTCTCTGCTGGCCGAGCTGCTGGTACGTTGGCACGTTGGCACGTTGGCTAACCCAACGCAAGCTGGTCCGGGAGGTATATCTGGCCCGTACATCGATCT-3’. The product was transformed into *E. coli* GS1783 containing the MCMV BAC. Following Kan^r^ cassette removal and homologous recombination, selected BAC clones were verified via restriction digestion, and the desired mutation was analyzed via Sanger sequencing. MCMV BAC DNA was purified using the NucleoBond BAC 100 kit (Macherey-Nagel #740579), followed by transfection into NIH-3T3 or m54.5p-V5-3T3 cells using the TransIT-X2 system (Mirus).

### Production of m54.5 antibody

A 6XHis-tagged m54.5 ORF was cloned into the IPTG-inducible pET22b (+) vector (Novagen) via In-fusion cloning (Takara Bio In-fusion HD Cloning Plus kit). The PCR product was generated by using WT MCMV BAC as a template using primers 5’-GTGGTGCTCGAGTGCGGCCGCCATGGGATACCGTTCTTCTTGA-3’ and 5’-CCAGCCGGCGATGGCCATGGATATGGCTAACCCAACGCAAG-3’. The cloned vector was transformed into *E.coli* BL21. m54.5p recombinant protein expression and isolation was performed as per the Qiagen QIAexpressionist. The isolated protein was purified via affinity chromatography on Ni-sepharose columns. Purified protein was then used to immunize BALB/c mice to isolate mouse monoclonal antibodies as described previously. Clones were tested using ELISA on purified m54.5p. The antibody candidates were tested for immunoblotting by probing for m54.5p expression on lysates of NIH-3T3 cells infected with WT MCMV or its m54.5p-null mutant, as well as for immunofluorescence (IF) studies using the m54.5p-V5-3T3-tet-on and parental NIH-3T3 cell lines.

### Generation of m54.5p-expressing cell lines

m54.5p-expressing constitutive cell lines tagged with either a 1X V5 or FLAG epitope at the C-terminus were generated by In-fusion cloning (Takara Bio In-fusion HD Cloning Plus kit) of the suitable PCR products into the pMSCV-puro^R^ backbone (digested with EcoRI and XhoI). PCR products were amplified using primers 5’-CCGGAATTAGATCTCTCGAGGCCACCATGGCTAACCC-3’ and 5’-TCCCCTACCCGGTAGAATTCTCATGTACTGTCCAGTCCCAG-3’ (V5 tag) or 5’-TCCCCTACCCGGTAGAATTCTCACTTATCATCGTCATCCTTGTAATC-3’ (Flag tag). The m54.5p-expressing doxycycline-inducible system to generate the m54.5p-V5-3T3-tet-on cells was generated by cloning a PCR product into the TET-ON pLT3GG system (lacking the mirE sequence) between BamHI and EcoRI restriction sites. The PCR product was generated by primers 5’-GTCGAGCTTGCGTTGGATCCATGGCTAACCCAACGCAAG-3’ and 5’-CAAGATAATTGCTCGAATTCTCATGTACTGTCCAGTCCCAGG-3’. All PCR products were generated using CloneAmpHiFi PCR premix (Takara Bio. 639298). The obtained plasmids were transformed into Takara Stellar competent cells. Correct clones were analyzed via restriction digestion and Sanger Sequencing. DNA was purified using the ZymoPURE II Plasmid Midiprep kit. Lentiviruses were generated by transfecting HEK293T cells (6-well plate) with one of the above plasmids along with pVSVg and psPax2 (obtained from Addgene) using TransIT-X2 system (Mirus). Lentivirus supernatant (1–2 ml) was harvested 48 hours’ post-transfection to transduce NIH-3T3 cells.

### Immunoblotting

Cells were lysed with 2X Laemmli sample buffer (Cold Spring Harbor protocols). Lysed samples were heated at 95°C/10 minutes. Tris-Glycine SDS-PAGE (8–12%) and wet transfer (Tris-Glycine-20% Methanol) on 0.2 μm Nitrocellulose membrane (Amersham Protran) were performed using the Mini Gel Tank (Life technologies). Membranes were subsequently subject to blocking in 5% (v/v) skimmed milk in 1X PBST (phosphate-buffered saline, 0.1% Tween 20) at room temperature for one hour. Membranes were probed with primary antibodies dissolved in 3% BSA-PBST (0.1% Tween-20), overnight at 4^o^C at the following concentrations: mouse anti-m54.5p (1:2), rabbit Anti-V5 tag (D3H8Q) mAb (1:1000, Cell Signalling Technology 13202S), rat Anti-DYKDDDDK (FLAG) epitope (L5) antibody (1:1000, Novus Biologicals NBP106712), mouse anti-m123/IE1 (MCMV) antibody – clone IE1.01 (1:1000, CAPRI HR-MCMV-12), mouse anti-M55/gB (MCMV) antibody – clone M55.02 (1:1000, CAPRI HR-MCMV-14), mouse anti-M112-113/E1 (MCMV) antibody – clone CROMA103 (1:1000, CAPRI HR-MCMV-07), mouse Beta Actin antibody (C4) (1:1000, SCBT sc-47778), rabbit α-Tubulin Antibody (1:1000; #2144 Cell Signaling Technology), APC1 Polyclonal antibody (1:1000, 21748–1-AP Proteintech), PPP6C Polyclonal antibody (1:1000, 15852–1-AP Proteintech), rabbit SAPS3 antibody (1:1000, NBP2–34049, Novus Biologicals), rabbit Anti-V5 tag (D3H8Q) mAb (1:1000, Cell Signalling Technology 13202S), mouse Anti-Cyclin A mAb (1:1000, Sigma-Aldrich C4710), rabbit Anti-PPP6C (A48274) pAb (1:1000, Proteintech 15852–1-AP), rabbit Anti-geminin pAb (1:1000, Proteintech 10802–1-AP), rabbit Anti-securin pAb (1:500, Proteintech 18040–1-AP), rabbit Anti-APC1 pAb (1:500, Proteintech 21748–1-AP), rabbit Anti-APC2 pAb (1:500, Proteintech 13559–1-AP), rabbit Anti-APC4 pAb (1:500, Proteintech 14129–1-AP), rabbit Anti-APC7 pAb (1:500, Proteintech 21761–1-AP), rabbit Anti-APC8 pAb (1:500, Proteintech 10683–1-AP). Membranes were then washed for 10 minutes twice in 1X PBST followed by incubation with secondary antibodies dissolved in 3% BSA-PBST (0.1% Tween-20), for 1 hour at RT at the following concentrations: goat Anti-rabbit IgG (Whole molecule – HRP conjugated) (1:10,000, Sigma Aldrich A0545), rabbit Anti-mouse IgG (Whole molecule – HRP conjugated) (1:10,000 Sigma Aldrich A9044), rabbit Anti-rat IgG (Whole molecule – HRP conjugated) (1:10,000 Sigma Aldrich A5795), IRDye 800CW Goat Anti- mouse IgG (1:1000, LI-COR Biosciences 92632210), IRDye 680RD Goat α-rabbit IgG (1:1000, LI-COR Biosciences 92668071). Followed by washing, proteins were analyzed by visualizing the membranes on LI-COR Odyssey FC Imaging System. Proteins bound by HRP-conjugated antibodies were developed using the Thermo Fischer SuperSignal West Pico PLUS substrates as per the company’s instructions.

### Co-immunoprecipitation

V5 Co-immunoprecipitation (Co-IP) samples were prepared from the m54.5p-expressing constitutive cell lines (V5/FLAG-tagged). For each sample, ten million cells were washed and collected by scraping cells in 1ml 1X ice-cold PBS. After centrifugation (1000G/10 minutes), cells were lysed in 1 ml IP lysis buffer (50 mM Tris-HCL (pH:7.4), 300 mM NaCl, 1% (w/v) Triton X-100, 1 mM EDTA, 1 mM PMSF, complete protease inhibitor cocktail (1 tablet/10 mL), Benzonase (50 units/mL, ChemCruz)) for 1 hour at 4^o^C with constant rotation. Lysed cells were then centrifuged at 18,000 G/15 minutes. The pellet was discarded and 50 μl of supernatant was collected as input. 950 μl was incubated with 25 μl of V5-Trap Magnetic Agarose beads (Chromotek) overnight at 4^o^C with constant rotation. Beads were magnetically separated using the DynaMag2 (Invitrogen) set up and supernatant was collected as the flow through fraction. Both input and flow through fractions were lysed in 4X Laemmli buffer (2X final concentration). Beads were washed 5 times in IP wash buffer (50 mM Tris-HCL (pH:7.4), 150 mM NaCl, 1% (w/v), complete protease inhibitor cocktail (1 tablet/10 mL)) and finally boiled in 100–200 μl 2X Laemmli buffer at 95^o^C for 10 minutes to extract proteins (output). Laemmli buffer without DTT was used to make samples for mass spectrometry. Co-IP samples were then subject to Immunoblotting for further analysis.

### Mass spectrometry

Samples lysed in 2X Laemmli buffer were incubated with 4x volume acetone at 20^o^C/overnight to precipitate proteins. Pellets were washed in acetone at -20^o^C. Precipitated proteins were dissolved in NuPAGE LDS sample buffer (Life Technologies), reduced with 50 mM DTT at 70°C for 10 minutes and alkylated with 120 mM iodoacetamide at room temperature for 20 minutes. Separation was performed on NuPAGE Novex 4–12% Bis-Tris gels (Life Technologies) with MOPS buffer according to manufacturer’s instructions. Gels were washed three times for 5 min with water and stained for 60 min with Simply Blu Safe Stain (Life Technologies). After washing with water for 1 h, each gel lane was cut into 15 slices. The excised gel bands were destained with 30% acetonitrile in 0.1 M NH_4_HCO_3_ (pH 8), shrunk with 100% acetonitrile, and dried in a vacuum concentrator (Concentrator 5301, Eppendorf, Germany). Digests were performed overnight at 37°C with 0.1 µg trypsin per gel band in 0.1 M NH_4_HCO_3_ (pH 8). After removing the supernatant, peptides were extracted from the gel slices with 5% formic acid, and the extracted peptides were pooled with the supernatant. NanoLC-MS/MS analyses were then performed on an Orbitrap Fusion (Thermo Scientific) equipped with a PicoView Ion Source (New Objective) and coupled to an EASY-nLC 1000 (Thermo Scientific). Peptides were loaded on a trapping column (2 cm x 150 µm ID, PepSep) and separated on a capillary column (30 cm x 150 µm ID, PepSep), both packed with 1.9 µm C18 ReproSil and separated with a 30-minute linear gradient from 3% to 30% acetonitrile and 0.1% formic acid and a flow rate of 500 nl/min. Both MS and MS/MS scans were acquired in the Orbitrap analyzer with a resolution of 60,000 for MS scans and 30,000 for MS/MS scans. HCD fragmentation with 35% normalized collision energy was applied. A Top Speed data-dependent MS/MS method with a fixed cycle time of 3 s was used. Dynamic exclusion was applied with a repeat count of 1 and an exclusion duration of 30 s; singly charged precursors were excluded from selection. Minimum signal threshold for precursor selection was set to 50,000. Predictive AGC was used with AGC a target value of 4x10^5^ for MS scans and 5x10^4^ for MS/MS scans. EASY-IC was used for internal calibration.

### Mitotic arrest assay and flow cytometry

Doxycycline-induced m54.5p-V5-3T3-tet-on cell lines and NIH-3T3 cells treated with or without Nocodazole (800 ng/ml) for 16 hours were washed in 1X PBS and harvested by trypsinization. Harvested cells were fixed and permeabilized in ice-cold 80% methanol and stored at 4^o^C for 20 minutes. Fixed cells were washed twice in 1X PBS and incubated with 50 µl RNAse A (100 µg/ml) and 200 µl propidium iodide solution (50 µg/ml) at 37^o^C (dark) for 30 minutes to stain DNA. Cells were directly strained and used for flow cytometry analysis using BD FACSCalibur. Briefly, NIH-3T3 Cells were gated via FSC vs SSC analysis, followed by segregation of single cells by FL-2 area (A) vs width (W) channels. The FL2-H channel was then utilized to study propidium iodide-stained cells and cell cycle.

### CellTrace Cell Proliferation

Doxycycline-induced m54.5p-V5-3T3-tet-on cell lines and NIH-3T3 cells were plated. CellTrace Violet dye was diluted in prewarmed 1X PBS to working concentration (5uM) immediately prior to use. The culture medium from the cells was removed and replaced with the loading solution. The cells were incubated for 20 minutes at 37°C. The cells were washed twice with culture medium and incubated in complete culture medium for 5 days. Harvested cells were fixed with 4% PFA and permeabilized in 0.2% TritonX-100. After fixation, cells were immunostained with an anti-V5 antibody (Rabbit Anti-V5 tag (D3H8Q) mAb, 1:1000, Cell Signaling Technology 13202S), followed by a secondary anti-rabbit IgG-Alexa Fluor 647 antibody. The samples were then analyzed by flow cytometry.

### Immunofluorescence

Seeded cells were washed in 1X PBS and fixed in 4% paraformaldehyde for 15 minutes at RT, followed by permeabilization in 0.5% Triton-X 100 for 5 minutes at RT. Cells were washed twice in 1X PBS followed by blocking in 10% FCS-1X PBS. Primary antibodies were diluted in 10% FCS-1X PBS in the following concentrations: Mouse Anti-m54.5p (1:2), Rabbit Anti-V5 tag (D3H8Q) mAb (1:1000, Cell Signalling Technology 13202S), Rat Anti-DYKDDDDK (FLAG) epitope (L5) antibody (1:1000, Novus Biologicals NBP106712). Cells were washed twice in 1X PBS and incubated with secondary antibodies (diluted in 10% FCS-1X PBS) in the following concentrations: Anti-Rabbit IgG (H + L) AlexaFluor 647 (1:1000, Thermo Fisher Scientific A21247), Anti-Rat IgG (H + L) AlexaFluor 488 (1:1000, Thermo Fisher Scientific A11006), Anti-Mouse IgG (H + L) AlexaFluor 568 (1:1000, Abcam ab175473). Cells were washed twice in 1X PBS and incubated with 1X DAPI solution for 5 minutes to stain nuclei before washing and processing. Microcopy was conducted using the Leica DMi8 (Leica Microsystems) device as per the manufacturers’ instructions.

### Confocal microscopy for subcellular localization analysis

Cells grown on glass coverslips were fixed with 4% paraformaldehyde for 15 minutes at RT, followed by permeabilization in 0.5% Triton-X 100 for 5 minutes at RT. Cells were washed twice in 1X PBS followed by blocking in 10% FCS-1X PBS. Primary antibodies were diluted in 10% FCS-1X PBS in the following concentrations: Rabbit Anti-V5 tag (D3H8Q) mAb (1:1000, Cell Signalling Technology 13202S), Mouse Anti-V5 tag (SV5-Pk1) mAb (1:500, Thermo Fisher Scientific R960-25), Mouse Anti-Cyclin A mAb (1:1000, Sigma-Aldrich C4710), Rabbit Anti-PPP6C (A48274) pAb (1:1000, Proteintech 15852–1-AP), Rabbit Anti-geminin pAb (1:1000, Proteintech 10802–1-AP), Rabbit Anti-securin pAb (1:500, Proteintech 18040–1-AP), Rabbit Anti-APC1 pAb (1:500, Proteintech 21748–1-AP), Rabbit Anti-APC2 pAb (1:500, Proteintech 13559–1-AP), Rabbit Anti-APC4 pAb (1:500, Proteintech 14129–1-AP), Rabbit Anti-APC7 pAb (1:500, Proteintech 21761–1-AP), Rabbit Anti-APC8 pAb (1:500, Proteintech 10683–1-AP). Cells were washed twice in 1X PBS and incubated with secondary antibodies (diluted in 10% FCS-1X PBS) in the following concentrations: Anti-Rabbit IgG (H + L) AlexaFluor 647 (1:1000, Thermo Fisher Scientific A21247), Anti-Mouse IgG (H + L) AlexaFluor 488 (1:1000, Thermo Fisher Scientific A21202). Cells were washed twice in 1X PBS and incubated with 1X DAPI solution for 5 minutes to stain nuclei before washing and processing. Images were acquired using a 100 X oil immersion objective on a wild-field fluorescence microscope (ZEISS) and processed using ZEISS ZEN.

### EdU labelling and click chemistry

NIH-3T3 cells were synchronized by serum starvation for 48 hours and subsequently were labelled with 10 µM EdU for a duration of 1 hour to label DNA-synthesizing cells. Following fixation and Permeabilization, click reaction was conducted using sodium ascorbate (10mM), CuSO_4_ (1mM), amino guanidine (10mM), and thiamine triphosphatase (THPTA (1mM)) along with clickable azide (488 nm – 10 µM) for 60 minutes at RT in the dark. Cells were then washed thrice in 1X PBS and processed for further staining (immunofluorescence) or microscopy.

### Data analysis and statistics

All immunoblotting data were analyzed on ImageStudio Lite (LI-COR). Flow cytometry analysis was performed on a CellQuestPro (BD Biosciences) for data acquisition. FlowJo 10 (BD Biosciences) was used for gating and histogram analysis. Sanger Sequencing data were analyzed on Snapgene (Dotmatics). Graphs were plotted using GraphPad Prism 9 (Dotmatics) unless otherwise stated. Student’s paired two-tailed parametric t-test was used to determine statistical significance and p-values. For MS data, raw MS data files were analyzed with MaxQuant version 1.6.2.2 [67]. Database searches were performed with Andromeda integrated in MaxQuant. The search was performed against the UniProt Mouse Reference Proteome database (March 2022, UP000000589, 21985 entries) and the UniProt Mouse cytomegalovirus Reference Proteome Database (March 2022, UP000122533, 164 entries). The sequence of the m54.5p was added to this database. Additionally, a database containing common contaminants was used. The search was performed with tryptic cleavage specificity with 3 allowed mis-cleavages. Protein identification was under control of the false discovery rate (FDR; < 1% FDR on protein and peptide spectrum match (PSM) level). In addition to MaxQuant default settings, the search was performed against the following variable modifications: Protein N-terminal acetylation, Gln to pyro-Glu formation (N-term. Gln) and oxidation (Met). Carbamidomethyl (Cys) was set as a fixed modification. Further data analysis was performed using R scripts developed in-house. LFQ intensities were used for protein quantitation. Proteins with less than two razor/unique peptides were removed. Missing LFQ intensities were imputed with values close to the baseline. Data imputation was performed with values from a standard normal distribution with a mean of the 5% quantile of the combined log10-transformed LFQ intensities and a standard deviation of 0.1. For the identification of significantly enriched proteins, median log2 transformed protein ratios were calculated from the three replicate experiments, and boxplot outliers were identified in intensity bins of at least 300 proteins. Log2 transformed protein ratios of sample versus control with values outside a 1.5x (significance 1) or 3x (significance 2) interquartile range (IQR), respectively, were considered as significantly enriched in the individual replicates. In addition, the R package limma was used to calculate Benjamini-Hochberg adjusted p-values and FDR [68,69].

### Use of AI tools

AI tools (Gemini, Google, and Grammarly) were used for literature search and language editing, as well as grammar and spelling checks. No AI tools were used for data analysis. The authors retain full responsibility for the manuscript.

## Supporting information

S1 File Multiple Sequence Alignment of m54.5 ORF from Diverse Mouse CMV Strains. Multiple sequence alignment of the m54.5 open reading frame (ORF) from various mouse cytomegalovirus (CMV) strains, generated using Clustal Omega and visualized with Jalview. The start codon (ATG) is marked in green, the stop codon (TGA) is shown in black, and all other nucleotides are color-coded according to purine (A/G) and pyrimidine (C/T) classification. This alignment illustrates both conserved and variable nucleotide regions among the analyzed strains.
(PDF)

S2 File Potential additional ORFs located within the highly conserved viral DNA polymerase genes of MCMV, HCMV, RCMV, and GPCMV. For each viral DNA polymerase gene sequence (MCMV M54, HCMV UL54, RCMV E54, and GPCMV GP54), the DNA was translated in all three forward reading frames. The canonical polymerase frame is displayed at the top, with the two alternative reading frames shown below, and a nucleotide length ruler is presented at the bottom of each panel. Potential open reading frames (ORFs), defined as regions extending from a start codon (AUG) to the next in-frame stop codon, are highlighted with a light-yellow background, and the number of amino acids (aa) encoded by each ORF is indicated. In the M54 frame, putative ORFs of 227, 119, and 73 aa were identified. Ribo-seq only confirmed the 227 aa m54.5 ORF; in UL54, putative ORFs of 98, 80, and 37 aa were identified; in the alternative frames of E54, putative ORFs of 114, 73, 55, and 39 aa were identified; and in GP54, putative ORFs of 54, 52, 43, and 42 aa were identified. Of note, no ORFs of relevant length were identified in the C-terminal parts of the M54 homologs, where the m54.5 ORF is located.
(PDF)

S3 File Comparative conservation of m54.5 protein sequences identified by tBLASTn. The protein sequence of MCMV m54.5p was used as a query in a tBLASTn search against the NCBI nucleotide database. The top 10 significant hits from different cytomegalovirus (CMV) organisms were retrieved as nucleotide sequences, translated into amino acid sequences using EMBOSS Transeq, and aligned with Clustal Omega. The resulting multiple sequence alignment was visualized in Jalview with the “Clustal” color scheme, highlighting conserved residues. Stop codons are indicated by white asterisks (*) on a black background. Sequence conservation across all aligned proteins is indicated.
(TIFF)

S4 File Independent biological replicates of Western blot experiments. (**A**) Second biological replicate of the experiment shown in Fig 1D. (**B**) Second biological replicate of the experiment shown in Fig 1E. (**C**) Second biological replicate of the experiment shown in Fig 2B. (**D**) Three independent biological replicates of the experiment shown in Fig 3B. (**E**) Second biological replicate of the experiment shown in Fig 3D.
(TIF)

S5 File Independent biological replicates of immunofluorescence and flow cytometry experiments. **(A**) Second biological replicate of the experiment shown in Fig 1C. (**B**) Second replicate of the experiment shown in Fig 2C. (**C**) Second biological replicate of the experiment shown in Fig 4B. (**D**) Second biological replicate of the experiment shown in Fig 4E. (**E**) Second biological replicate of the experiment shown in Fig 4I.
(TIF)

S6 File Control immunostaining for APC/C components and cell cycle substrates related to Fig 5. Confocal microscopy of m54.5p-V5-3T3-tet-on and control NIH-3T3 cells ± 24 h doxycycline. Cells were immunostained for the indicated APC/C components (APC1, APC2, APC4, APC7, APC8), PP6C, and APC/C substrates (geminin, securin, and Cyclin A). DAPI stains cell nuclei. Results include NIH-3T3 controls to complement data shown in Fig 5.
(TIF)

S7 File Western blot analysis of APC/C components and cell cycle substrates upon ectopic m54.5p expression. Western blot analysis for the indicated proteins (APC1, APC2, APC4, APC7, APC8), PP6C, and APC/C substrates (geminin, securin, and Cyclin A) was performed on whole-cell lysates from m54.5p-V5-3T3-tet-on cells and control NIH-3T3 cells with or without 24h of doxycycline induction. Β-actin served as a loading control. Note that securin and APC8 were undetectable under these conditions. Data from two independent experiments are shown.
(JPG)

S1 Table Table of the top interactors of m54.5. Both p-values of the interaction significance and fold change (V5/FLAG) are shown (data of 3 biological replicates) for both uninfected and infected conditions.
(XLSX)
