# Supplementary material for: A murine cytomegalovirus cell cycle regulator (m54.5p) evolved within the conserved viral DNA polymerase gene
Source: PLoS Pathog. 2026 May 22;22(5):e1013424. doi: 10.1371/journal.ppat.1013424 (PMC13229380; doi:10.1371/journal.ppat.1013424)
Supplement: S1 File — Multiple sequence alignment of the m54.5 open reading frame (ORF) from various mouse cytomegalovirus (CMV) strains, generated using Clustal Omega and visualized with Jalview. The start codon (ATG) is marked in green, the stop codon (TGA) is shown in black, and all other nucleotides are color-coded according to purine (A/G) and pyrimidine (C/T) classification. This alignment illustrates both conserved and variable nucleotide regions among the analyzed strains. (PDF) [file ppat.1013424.s001.pdf]

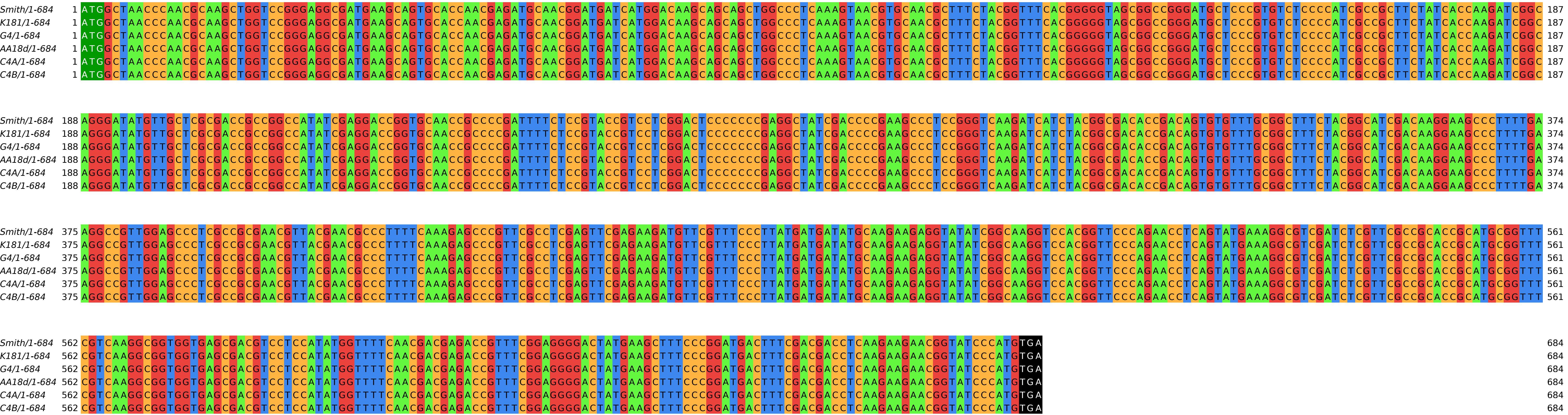
Smith/1-684
K181/1-684
G4/1-684
AA18d/1-684
C4A/1-684
C4B/1-684
1
187
188
374
375
561
562
684
