# Supplementary figures and images for: A murine cytomegalovirus cell cycle regulator (m54.5p) evolved within the conserved viral DNA polymerase gene
Source: PLoS Pathog. 2026 May 22;22(5):e1013424. doi: 10.1371/journal.ppat.1013424 (PMC13229380; doi:10.1371/journal.ppat.1013424)

MCMV M54

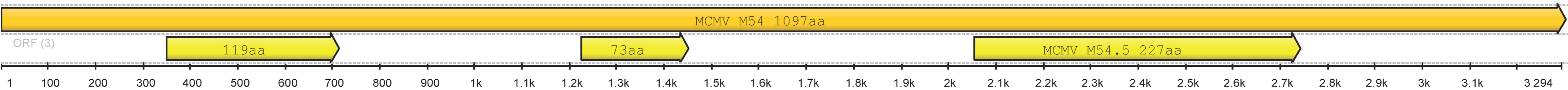

HCMV UL54

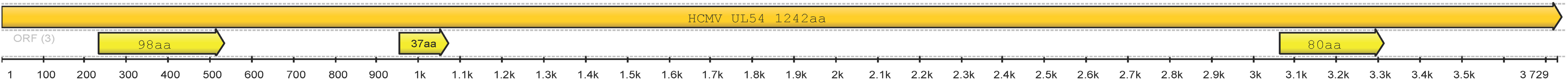

RCMV E54

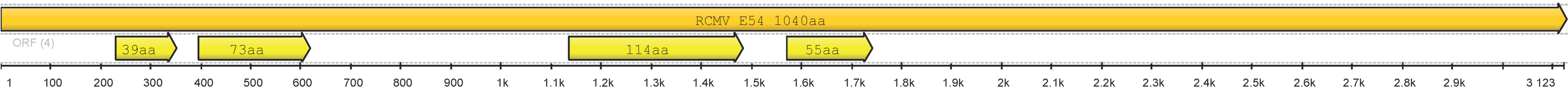

GPCMV GP54

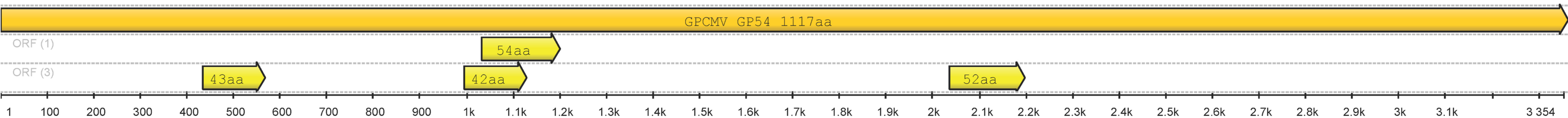

Supplement: S2 File — For each viral DNA polymerase gene sequence (MCMV M54, HCMV UL54, RCMV E54, and GPCMV GP54), the DNA was translated in all three forward reading frames. The canonical polymerase frame is displayed at the top, with the two alternative reading frames shown below, and a nucleotide length ruler is presented at the bottom of each panel. Potential open reading frames (ORFs), defined as regions extending from a start codon (AUG) to the next in-frame stop codon, are highlighted with a light-yellow background, and the number of amino acids (aa) encoded by each ORF is indicated. In the M54 frame, putative ORFs of 227, 119, and 73 aa were identified. Ribo-seq only confirmed the 227 aa m54.5 ORF; in UL54, putative ORFs of 98, 80, and 37 aa were identified; in the alternative frames of E54, putative ORFs of 114, 73, 55, and 39 aa were identified; and in GP54, putative ORFs of 54, 52, 43, and 42 aa were identified. Of note, no ORFs of relevant length were identified in the C-terminal parts of the M54 homologs, where the m54.5 ORF is located. (PDF) [file ppat.1013424.s002.pdf]

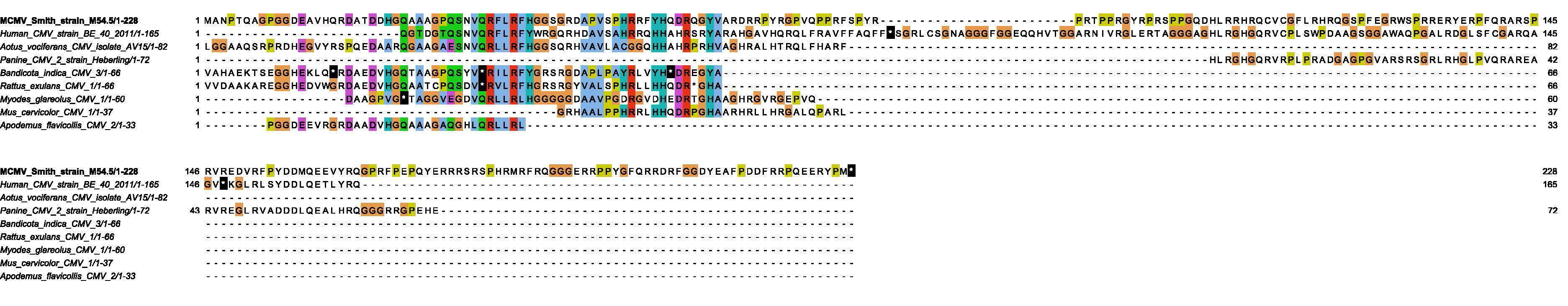

Supplement: S3 File — The protein sequence of MCMV m54.5p was used as a query in a tBLASTn search against the NCBI nucleotide database. The top 10 significant hits from different cytomegalovirus (CMV) organisms were retrieved as nucleotide sequences, translated into amino acid sequences using EMBOSS Transeq, and aligned with Clustal Omega. The resulting multiple sequence alignment was visualized in Jalview with the “Clustal” color scheme, highlighting conserved residues. Stop codons are indicated by white asterisks (*) on a black background. Sequence conservation across all aligned proteins is indicated. (TIFF) [file ppat.1013424.s003.tiff]

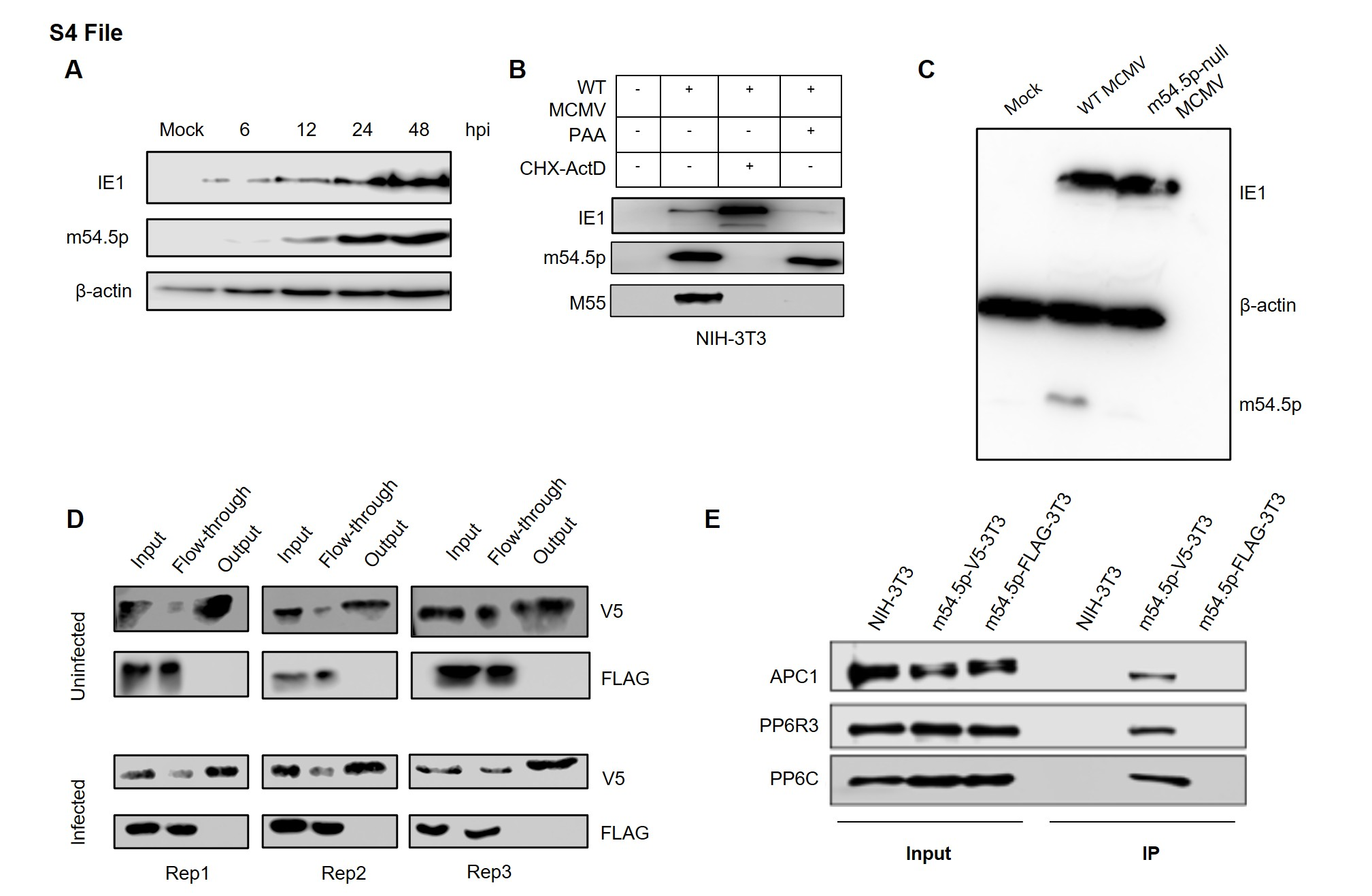

Supplement: S4 File — (A) Second biological replicate of the experiment shown in Fig 1D. (B) Second biological replicate of the experiment shown in Fig 1E. (C) Second biological replicate of the experiment shown in Fig 2B. (D) Three independent biological replicates of the experiment shown in Fig 3B. (E) Second biological replicate of the experiment shown in Fig 3D. (TIF) [file ppat.1013424.s004.tif]

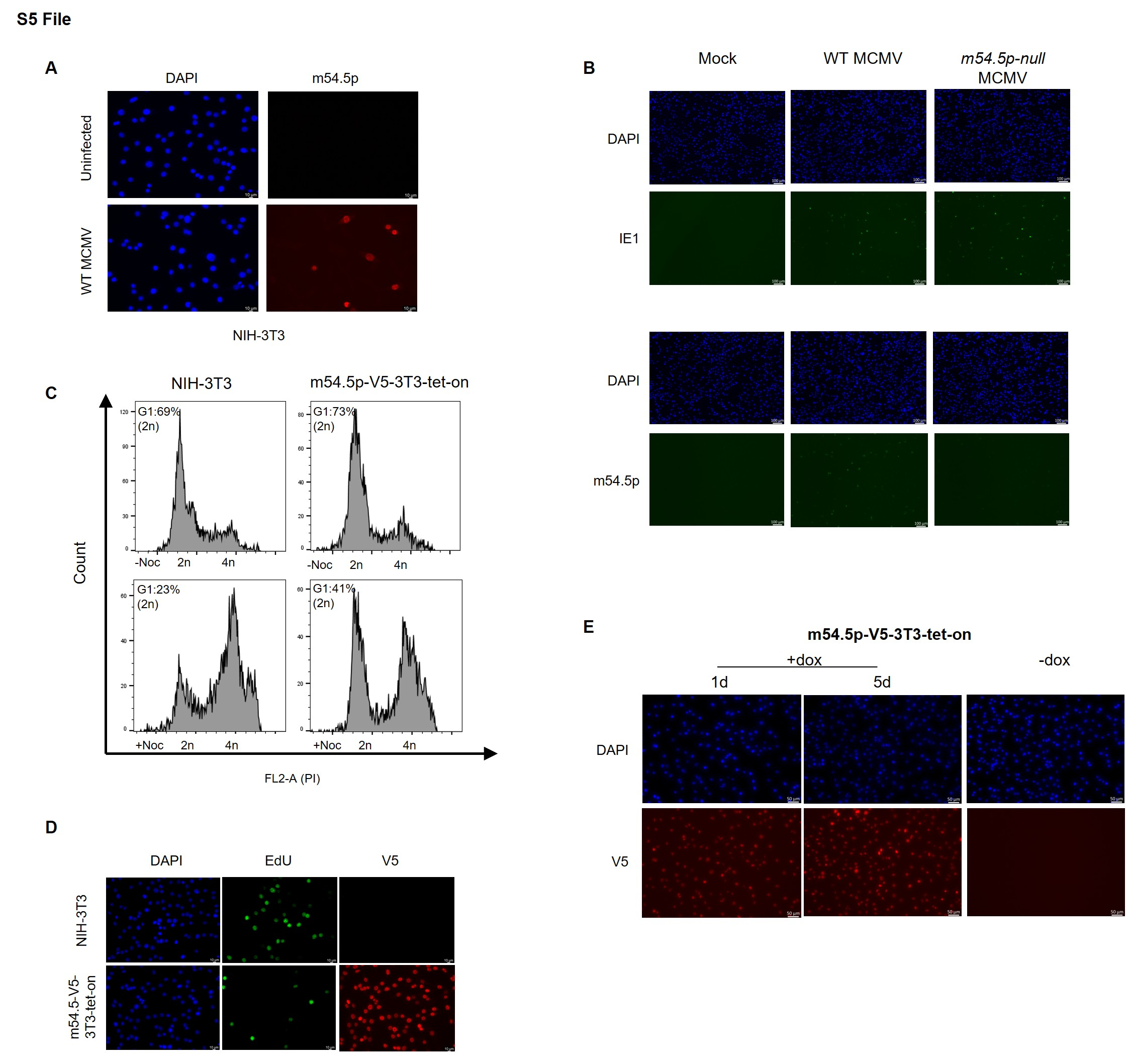

Supplement: S5 File — (A) Second biological replicate of the experiment shown in Fig 1C. (B) Second replicate of the experiment shown in Fig 2C. (C) Second biological replicate of the experiment shown in Fig 4B. (D) Second biological replicate of the experiment shown in Fig 4E. (E) Second biological replicate of the experiment shown in Fig 4I. (TIF) [file ppat.1013424.s005.tif]

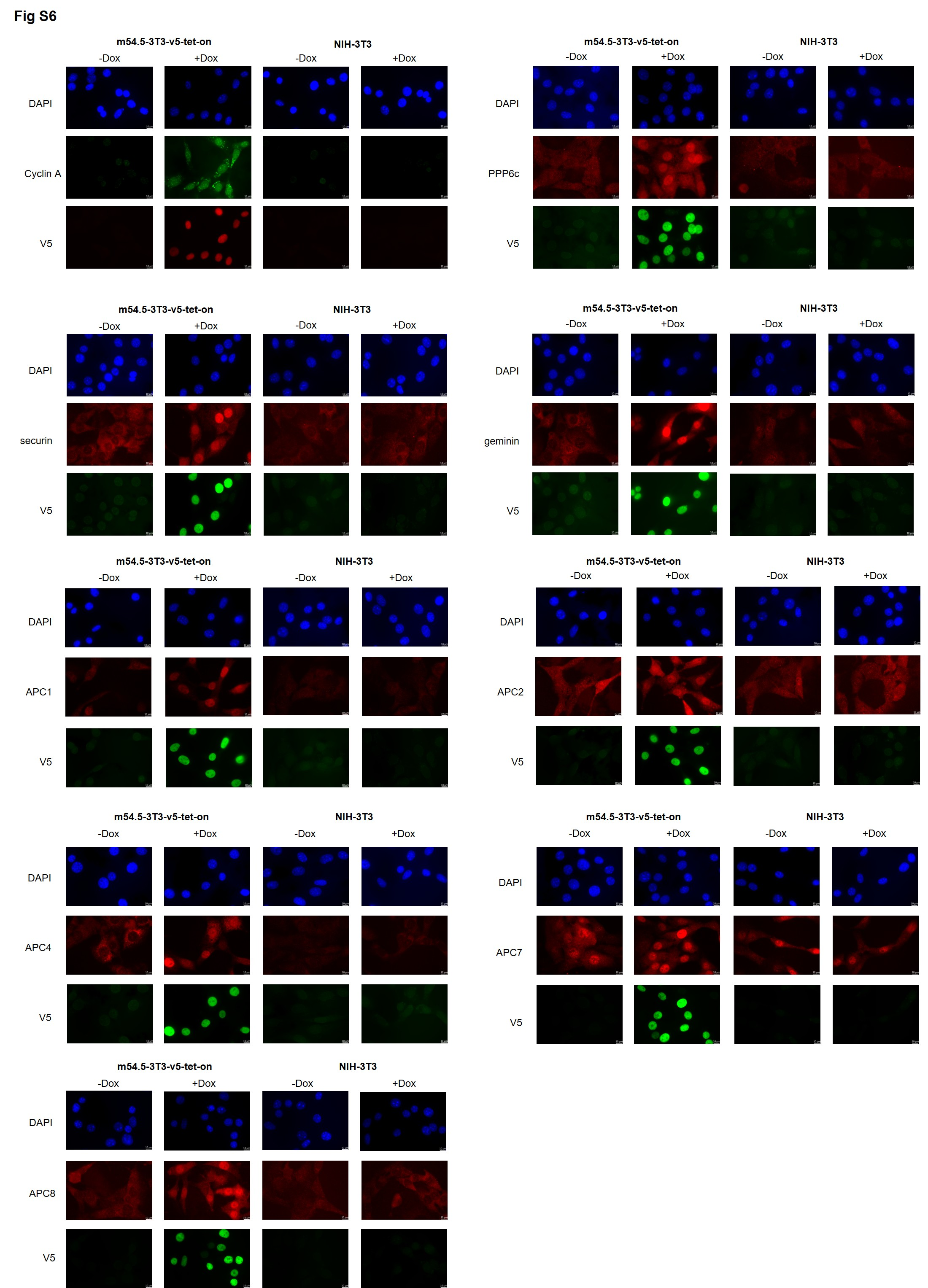

Supplement: S6 File — Confocal microscopy of m54.5p-V5-3T3-tet-on and control NIH-3T3 cells ± 24 h doxycycline. Cells were immunostained for the indicated APC/C components (APC1, APC2, APC4, APC7, APC8), PP6C, and APC/C substrates (geminin, securin, and Cyclin A). DAPI stains cell nuclei. Results include NIH-3T3 controls to complement data shown in Fig 5. (TIF) [file ppat.1013424.s006.tif]

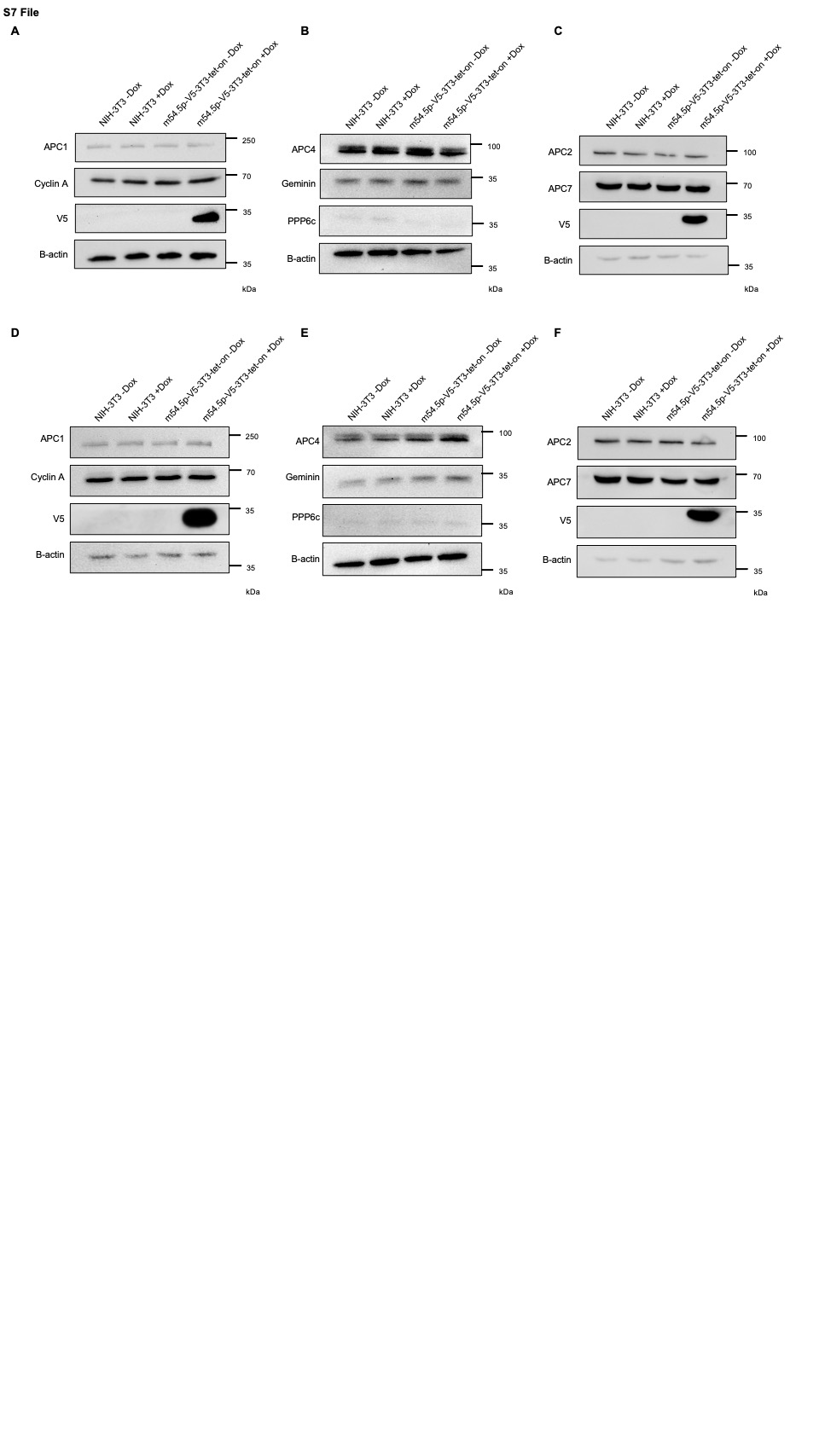

Supplement: S7 File — Western blot analysis for the indicated proteins (APC1, APC2, APC4, APC7, APC8), PP6C, and APC/C substrates (geminin, securin, and Cyclin A) was performed on whole-cell lysates from m54.5p-V5-3T3-tet-on cells and control NIH-3T3 cells with or without 24h of doxycycline induction. Β-actin served as a loading control. Note that securin and APC8 were undetectable under these conditions. Data from two independent experiments are shown. (JPG) [file ppat.1013424.s007.jpg]
